# On the Importance of the Starting Material Choice and Analytical Procedures Adopted When Developing a Strategy for the Nanoencapsulation of Saffron (*Crocus sativus* L.) Bioactive Antioxidants

**DOI:** 10.3390/antiox12020496

**Published:** 2023-02-16

**Authors:** Maria Z. Tsimidou

**Affiliations:** Laboratory of Food Chemistry and Technology, School of Chemistry, Aristotle University of Thessaloniki (AUTH), 54124 Thessaloniki, Greece; tsimidou@chem.auth.gr

**Keywords:** saffron, *Crocus sativus* L., nanoencapsulation, crocins, crocetin, picrocrocin, safranal, saffron extracts, saffron antioxidants, saffron analysis

## Abstract

Saffron is known as the most expensive spice in the world. It is comprised of the dried stigmas of the pistil of the *Crocus sativus* L., which is a cultivated, sterile crocus plant. This plant material is now recognized as the unique edible source of certain bioactive apocarotenoids for which in-vivo antioxidant properties have been reported. Among the latter, crocins, red-orange natural colorants, and their parent molecule crocetin prevail in bioactivity significance. This review is focused on the strategies developed so far for their nanoencapsulation in relation to the characteristics of the starting material, extraction procedures of the bioactive antioxidants and analytical methods applied for their characterization and quantification throughout the process. The literature so far points out gaps that lead to publishable data, on one hand, but not necessarily to repeatable and meaningful processes due to incomplete characterization of the starting and the released material in efficiency and stability studies of the nanoencapsulates. Accurate terminology and quantitative chromatographic or spectrophotometric procedures for the determination of the core compounds are needed. Authenticity control and quality of saffron samples, and the verification of the concentrations of compounds in commercial preparations labeled as ‘crocin,’ are prerequisites in any experimental design setup.

## 1. Saffron as a Source of Bioactive Antioxidants

**Figure 1:**
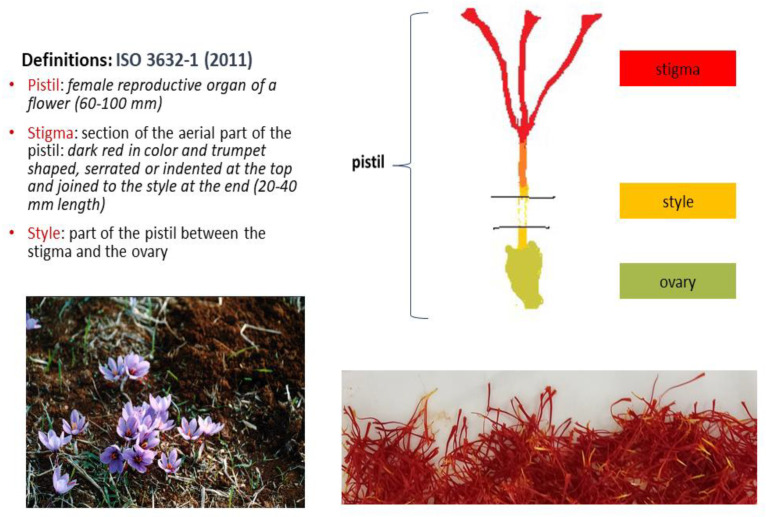
Photos of saffron plants and pistils (M.Z.T. personal collection), female organ and definitions of traded plant material according to the relevant ISO standard [1].

*Crocus sativus* L. is a cultivated, sterile crocus plant. It is the exclusive edible source of saffron, the most expensive spice in the world. The three-branch stigmas on the upper aerial part of its pistil after drying comprise the spice (Figure 1), which is traded in filaments (whole or cut) or powder form as three different commercial categories [1], in bulk or packed in different quantities according to buyer and seller agreements. The commercial categories of saffron are distinguished on the basis of quality criteria (% extraneous matter, % foreign matter, % moisture content, % ash content, % acid-insoluble ash, % soluble extract in cold water, coloring, flavor and aroma strengths) determined according to test methods described in a previous study [2]. The values for the latter three parameters are derived spectrophotometrically at 440 nm (λ_max_ for crocins), 257 nm (λ_max_ for picrocrocin) and 330 nm (λ_max_ for safranal). Saffron is considered the richest edible source of these groups of rare bioactive apocarotenoids. In particular, saffron is rich in the colored group of crocins, which are tetraterpenoid compounds bearing seven conjugated double bonds. Their parent molecule is crocetin but it is not naturally present in its free form. Saffron is also rich in picrocrocin, a colorless bitter glycoside whereas, among the many volatiles present in its essential oil, the hydrophobic safranal is reported as the most characteristic [3,4].

The *trans*-crocetin di *(β*-D-gentiobiosyl) ester (or crocin 1) is the most abundant member of crocins, often mentioned in the literature under the misleading trivial name ‘crocin’. Saffron aqueous and aqueous methanol extracts are rich in crocins and picrocrocin (e.g., mean value 33.5 ± 4.30 and 15.3 ± 3.1 g/100 g dry stigmas, *n* = 22, Greek saffron grade I, respectively) [5]. Picrocrocin is more hydrophilic than crocins. Their content varies among saffrons of different commercial grades [1] and is influenced by extraction procedures [4,6,7]. On the other hand, the volatile safranal is more hydrophobic and it is better extracted using permitted organic solvents of low boiling point beyond methanol [8,9]. Its content determined by gas chromatography was found to vary considerably among commercial samples (0.54–10.7 g/kg dry saffron, *n* = 76) [9]. There is a continuous interest in testing the effectiveness of new means of extracting saffron bioactive compounds, which can be more operative, greener and cost effective (see [10] and references therein).

The basic structures and the IUPAC nomenclature of saffron bioactive compounds are shown in Table 1. Trivial names for crocins are not preferred to avoid mistakes often found in the literature due to the use of obsolete or incorrect interpretations of the structural characteristics.

As applicable for most spices, producing countries are usually only few and different from those which add value to finished products and control the retail market by re-exporting. Iran prevails in the world production (~2–3 hundred tons annually). Greece prevails in the European Union as a producing country (~1 ton), whereas Spain plays a leading role in the trade of saffron originating from different countries [3,4].

Statistics for saffron market value are not easy to comprehend or verify. Data seem to be complicated, though indicative of a large variability in prices (USD per kilogram) which, for instance, for Iranian saffron range from USD 8.84 (China Macao market) to more than USD 2000 (Ukraine market) [11]. Saffron is found among the most frequently adulterated spices ([12] and the large variability in prices raises concerns about the integrity of the traded products. Authenticity criteria and methods of the detection of fraudulent practices are rather limited in the ISO trade standard 3632 [1,2] although the literature since 2000 has been steadily enriched in more effective methods to combat fraud [13], as is pointed out in Table 2 (columns 2 and 5). Research interest in the health properties of saffron bioactive compounds supersedes the interest in authenticity studies, as is also illustrated in Table 2 (columns 3 and 6), meaning the characterization of saffron as a functional spice to be strongly justified [3]. Saffron, saffron extracts, crocins, crocetin, safranal and picrocrocin have been studied for a variety of positive health effects such as antigenotoxic, antioxidant, antitumor, anti-inflammatory, antiatherosclerotic, antidiabetic, hypotensive, hypoglycemic, antihyperlipidemic, hepatoprotective, antidegenerative (central nervous system, retinal dysfunction) and antidepressant effects [4,14,15]. Crocins and, mainly, crocetin, are considered as in-vivo antioxidants acting against reactive oxygen species (ROS) conferring, thus, protections against oxidative stress. The antioxidant activity of these compounds and that of safranal is related to the reduction in lipid peroxidation (malondialdehyde levels) and nitric oxide levels, and the increase in the levels of glutathione antioxidant enzymes (superoxide dismutase, catalase, glutathione peroxidase) and thiol content [14,15]. The in-vivo activities are not well-understood yet, but the ongoing efforts of scientists to investigate their mechanisms have increased dramatically in the last decade, as is shown in Table 2.

All of these bioactive unsaturated compounds are prone to changes under different conditions of processing and storage. Processing which includes mainly the processes of drying and cleaning the stigmas using traditional practices affect their initial content and future degradation rate [16,17,18]. Degradation kinetics have been mainly studied for total or individual crocins in the dry matter [18,19,20], in aqueous extracts [21,22,23], in the presence of food additives [24,25] or under gastrointestinal conditions [26,27]. In dry forms, water activity is becoming the crucial parameter [18,19,20]. In aqueous extracts, pH values around 6.5–7 are the recommended region for slower degradation rates [21,22,23]. In all cases, processing and storage in the dark favor the stability of these apocarotenoids. Ambient temperatures and even storage at 4 °C are recognized as positive means to lengthen the shelf -life of the product and its extracts. Hypotheses that relate picrocrocin degradation to safranal-content increase have been revised over the years [18]. The application of more advanced analytical techniques supported more evidenced-based knowledge on the formation, degradation and interrelationships among the various bioactive compounds, from their biosynthesis [28,29] to changes due to post-harvest practices and fate during storage.

## 2. Encapsulation of Saffron Bioactive Compounds

**Figure 2:**
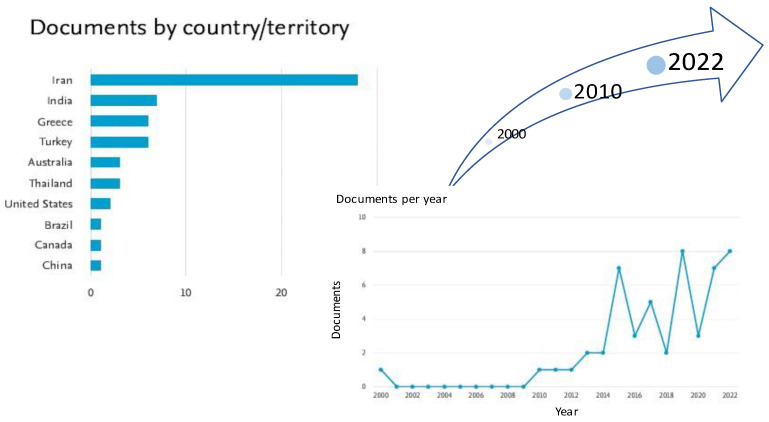
Graphical representation of the increasing number of publications per country since 2000 using as keywords saffron and encapsulation in the title, abstract, or keyword fields (51 documents of all types, Scopus search carried out in January 2023).

Though encapsulation has been applied in the food industry for more than half a century, to our knowledge, the first effort to protect the degradation of saffron bioactive crocins through encapsulation in the 21st century dates back to 2000 [30]. In that work, the authors aimed to shed light on the degradation kinetics of crocins in a saffron extract encapsulated in three amorphous polymer matrices (pullulan and two polyvinylpyrrolidone, PVP, materials differing in their molecular mass) using freeze drying. They examined whether the degradation rates of encapsulated saffron apocarotenoids depend on water activity or on the molecular mobility associated with the glass transition of the amorphous matrix. Efforts since 2000 have been published by different research groups, among which Iranian institutions prevail, as is shown in Figure 2.

Publications concern the encapsulation of saffron extracts per se or in combination with other compounds, and include typical technological aspects (the optimization of core–wall ratios, characterization of the nanoencapsulates, efficiency and stability studies) or also cover bioaccessibility and bioactivity issues. Focus is placed on nanoscale encapsulation [31]. Besides the above-mentioned groups of bioactive compounds, others, such as anthocyanins from the tepals of the flower, attracted the interest of certain researchers, but those studies will not be discussed in the present review.

At this point, it is worth stressing that both crocins and picrocrocin contents account for more than 40% of the dry weight of the spice. This value is much higher than those observed for other bioactive compounds in precious plant materials such as green and black tea (total polyphenols content, 14–21% and 8–18%, *w*/*w*, respectively) [32]. Therefore, it is important to ensure the maximum recovery of these compounds from the plant material prior to deciding which encapsulation technology or wall materials will be tested.

All types of encapsulation and nanoencapsulation technologies, in particular, have been applied so far in the relevant research papers to protect saffron extracts or specific compounds, i.e., crocins, crocetin, picrocrocin or safranal [30,33,34,35,36,37,38,39,40,41,42,43,44,45,46,47,48,49,50,51,52,53,54,55], and have been reviewed in detail, recently [7,56]. To our knowledge, the encapsulation of saffron bioactive compounds at the nano-scale appeared in 2013 and, since then, applications have been either for food and medical uses or aimed at a more generic target such as the enhancement of bioavailability. Nanoencapsulation strategies have found applications mainly for hydrophobic bioactive compounds such as phenolic compounds, carotenoids, essential oils, essential fatty acids, and insoluble vitamins, aiming at enhancing their low bioavailability and in-vivo stability beyond other technologically desirable effects such as resistance to pH, temperature, and masking of unpleasant flavor, etc. [57]. Nevertheless, only few of the numerous ideas found their way into the market due to the various preparation parameters that should be considered and optimized. The same precautions apply for saffron bioactive compounds, which are rather hydrophilic (crocins, picrocrocin), or fairly apolar (crocetin, safranal).

Since other preparation parameters have been recently discussed [7,56], this review highlights the strategies developed for the encapsulation of saffron bioactive compounds in relation to the characteristics of the starting material, extraction procedures and the analytical methods applied for the identification and quantification of the bioactive compounds throughout the process. These parameters have not been discussed carefully so far and can play a critical role in successful application. The review aims to support a standard methodology that will lead to obtaining more reliable data regarding the characteristics of the starting materials and the determination of the concentrations of bioactive compounds throughout the experimental steps (starting material, preparation of bioactive compound extracts or solutions, release studies, efficiency and stability studies) irrespective of the encapsulation technologies applied. Authenticity and quality control of the starting material is a prerequisite in the standardization of finished plant products. Standardized extracts can be further incorporated, encapsulated or not, in food, biological and cosmetic applications.

## 3. Choice of the Starting Material and Analytical Procedures Adopted

In most of the research papers, saffron was used as the starting material [30,35,36,37,39,40,43,46,48,49,50,51,52,53,54]. Commercially available products, under the trade name ‘crocin’, ‘crocin I’ or ‘crocin 4,′ from different suppliers or other individual compounds were used as the starting material in the rest of the studies [33,34,38,41,42,44,45,47,55]. Obviously, in the former case, all of the bioactive compounds of saffron can be potentially encapsulated whereas in the second case, focus is on targeted compounds (e.g., safranal, picrocrocin, crocetin or crocins). The list of references [30,33,34,35,36,37,38,39,40,41,42,43,44,45,46,47,48,49,50,51,52,53,54,55] is numbered according to the date they were published or became available on line. Table 3 and Table 4 summarize all the relevant data for the starting material and the analytical methods used for the characterization of the target compounds throughout the process of encapsulation, release studies, stability studies and bioavailability studies.

### 3.1. Saffron Samples as Starting Material

Table 3 presents the published work on nanoencapsulation efforts for saffron bioactive compounds with saffron as the starting material.

**Table 3:** En(nano)capsulation approaches for saffron bioactive antioxidants with focus on the characteristics of saffron as the starting material, extraction and analytical methods for their isolation and determination.

Reference	Extraction Procedure	Analytical Methods
[30]	The saffron sample was a representative mixture of saffron stigmas (harvest 1998), which was kindly donated by the Cooperative of Saffron Producers (Crocos, Kozani, Greece). The sample was air-dried in the dark and kept in a desiccator at 4 °C before use. Stigmas were used for the preparation of aqueous extracts within a month from the production date. Saffron (12 g) was extracted with 500 mL H2O under continuous shaking in an incubator at 25 °C for 16 h. The extract was filtered and freeze-dried and the freeze-dried powders were stored in the dark at −18 °C until use.	Coloring strength degradation expressed as E1% 440 nm, where E = A440n/C(1 g/100 mL) according to ISO 3632-2, 1993 [2].
[35]	Saffron powder (4 g) (Novin Saffron, Iran) was suspended in 50% aqueous ethanol (25 mL) and mixed for 2 min. The mixture was then centrifuged at 4000 rpm (2600× *g*) for 10 min to eliminate plant residues and the supernatant was separated. Another 25 mL of the 50% aqueous ethanol solution was added to the sediment and the extraction was repeated. This process was repeated six more times. The collected supernatant was then kept in a dark container at 4 °C until further use.	Crocin, safranal and picrocrocin were determined by direct reading the absorbance of 1% aqueous solution of saffron at 440, 330 and 257 nm, respectively, according to ISO 3632-2 [2].
[36]	Authentic Greek saffron (harvest year 2012) was donated by the Saffron Cooperative of Crokos (Kozani, Greece). Saffron stigmas (grade I), were ground in an agate mortar and passed through a 0.4 mm sieve just before further use. Samples were subjected to ultrasonication for various time periods at 0.2 duty cycles (active intervals, s) and 100% amplitude. The immersion depth of the probe was 20 mm. Sample temperature was kept at 15 ± 0.5 °C in a thermostated water bath. Appropriate amount of saffron (0.011–0.11 g) was added into a 50 mL Falcon tube, and then 20 mL of a methanol–water mixture (1:1, *v*/*v*) were added, according to [6]. An unblocked full factorial central composite design (CCD) was applied to study the effect of saffron–solvent ratio, *w/v*, and duration of sonication (min). Optimum conditions: 1:182 (*w/v*); sonication duration 29 min. Trans-crocetin di (β-D-gentiobiosyl) ester was isolated by semi-preparative RP-HPLC on a Nucleosil 100 C18 (250 × 10 mm i.d.; 7 μm) chromatographic column. The gradient elution system used consisted of water (A) and methanol (B). The gradient was: 0 min, 30% (B); 0–10 min, 45% (B); 10–20 min, 70% (B); 20–30 min, 100% (B); 30–40 min, 100% (B); and 40–50 min, 30% (B), and the flow rate was 3.0 mL/min. Monitoring was at 440 nm. Purity (97%) was checked (a) chromatographically by RP-HPLC-DAD in the range of 200–550 nm and calculated as the percentage of the total peak area at 440 nm and (b) by nuclear magnetic resonance (NMR) spectroscopy, recording the 1H 1D spectra at 300 MHz. Crocetin was precipitated from a saffron extract prepared under the optimum extraction conditions, after the acid hydrolysis of crocetin esters. Saffron powder (0.11 g) was weighted into a Falcοn tube to which 20 mL of a methanol:water mixture (1:1, *v*/*v*) were added. The mixture was sonicated for 29 min and finally centrifuged at 4100× *g* at 4 °C for 15 min. Methanol was evaporated under vacuum (40 °C), and the aqueous supernatant was acidified to pH 0.10 (± 0.03) by the addition of concentrate sulfuric acid solution, heated at 90 °C for 30 min, cooled, and centrifuged again under the same conditions. Hydrolysis of crocetin esters was monitored by TLC (development system, petroleum ether:acetic acid, 1:1, *v*/*v*) and HPLC of the hydrolysate. Crocetin was precipitated as a red powder. Residual crocetin esters were removed with repeated washes of the precipitate with deionized water (at least 3 × 100 mL) until the aqueous phase became colorless. Crocetin was then lyophilized. Its identity and purity were confirmed by UV-Vis spectroscopy, RP-HPLC-DAD, FT-IR and NMR spectroscopy.	Crocetin and crocetin esters were determined by HPLC-DAD. Separation was carried out on a LiChroCART Superspher 100 C18 (125 × 4 mm i.d.; 4 μm) column. The elution system used consisted of a mixture of water–acetic acid (1%, *v*/*v*) (A) and acetonitrile (B). The linear gradient was 20 to 100% (B) in 20 min. The flow rate was 0.5 mL/min. The analytical sample was prepared after proper dilution and filtration through a 0.45 μm membrane filter. Monitoring was in the range of 200–550 nm, and quantification of crocetin esters was carried out through the integration of the peak areas at 440 nm. Quantification of total crocetin esters content (g/100 g dry stigmas) was accomplished with the aid of a calibration curve of *trans-*crocetin di (β-D-gentiobiosyl) ester within the range 27.5–475 ng/10 μL injected volume (y = 38683x − 710440; R^2^ = 0.99; *n* = 7). Measurement repeatability was checked. During efficiency studies, crocetin quantification was accomplished with the aid of a calibration curve of isolated crocetin within the range 10–197 ng/10 μL injected volume (y = 14326x − 29306; R^2^ = 0.99; *n* = 5).
[37]	Dried stigmas of saffron were provided directly from the Cooperative of saffron, Crokos Kozanis. Saffron (1 g) was extracted with distilled water (50 mL) under continuous shaking in an ultrasound water bath at T = 25 °C for 60 min and at a fixed frequency of 30 kHz; saffron aqueous extracts were then filtered and kept in the dark at −30 °C until used.	The degradation of natural pigments was expressed as coloring strength (E) and followed by periodic absorbance measurements of the reconstituted powder (0.2 g) in aqueous solution with distilled water (10 mL, stirring for 10 min) and immediate measurement of the absorbance at 440 nm, the maximum absorption wavelength of crocin. The coloring strength was calculated as E1^%^ [2]
[39]	Saffron was picked before sunlight from a farm around Torbat- E Heydariyeh (Iran). Stigmas were separated from the other parts of the flowers and processed using different methods: (i) drying at room temperature (25 °C ± 1), (ii) dehydration with electrical oven (60 °C ± 1) and (iii) microwave drying (1000 W). Dried stigmas were crushed and sieved (0.421 mesh). Saffron powder was kept in an air-tight plastic bag within a desiccator at room temperature to prevent moisture absorption until used. Saffron extract was prepared through the extraction of dried powdered stigmas in water: 50% *v*/*v* ethanol for 2 h. The ratio of solvent to saffron powder was kept at a weight ratio of 100:1. The extract was filtrated (Whatman filter paper No. 42) and then concentrated in a rotary evaporator for about 30 min until 90% of the solvent was removed and stored at 4–5 °C before further use.	Picrocrocin, safranal and crocin content of saffron extract was determined using a UV-Vis spectrophotometer by measuring the absorbance at 257, 330 and 440 nm, respectively, as described by Orfanou and Tsimidou [58].
[40]	Saffron powder (10 g) was mixed with water (150 mL) in a dark colored bottle and incubated under shaking for 24 h. A rotor–stator homogenizer (10,000 rpm × 10 min) was used for maximum extraction of saffron bioactive compounds; the extract was filtered under vacuum, and kept in the freezer at −18 °C until use.	Spectrophotometry, expression of results for coloring strength (440 nm), bitterness (250 nm) and aroma (330 nm) according to ISO 3632 procedure [2].
[43]	As in [40]	As in [40]
[46]	Saffron was purchased as described in [6]. Ultrasound-assisted extraction of crocins and picrocrocin was according to [6]. Other conditions were as described in [36]. The optimum saffron–solvent ratio 1:182 (*w*/*v*) and sonication duration was 29 min, as according to [6]. Picrocrocin was laboratory-isolated according to [59]. Purity (99%) was checked as described in [6].	Crocins and picrocrocin in aqueous saffron extracts and in the obtained nanoencapsulates were determined using RP-HPLC-DAD and were quantified using external calibration curves (trans-crocetin di (β-D-gentiobiosyl) ester picrocrocin). Spectrophotometric monitoring of crocins and picrocrocin in stability studies was carried out in the region 200–600 nm. Quantification of total crocins and picrocrocin was accomplished using the calibration curves reported in [6].
[48]	Saffron was purchased from the Abbaszadeh Co. (Mashhad, Iran), Bioactive compounds of saffron were extracted through the aqueous extraction in [37,40] with some modifications. Briefly, saffron (1 g) was extracted with deionized water (20 mL) in a dark colored bottle and placed in a shaking incubator at ambient temperature for 24 h. The solution was treated by ultrasound with a power of 250 W and frequency of 30 kHz for 8 min at 25 °C. Aqueous saffron extract was filtered under vacuum using filter paper (Whatman No. 42) and frozen at −18 °C.	The released saffron extract based on safranal was determined spectrophotometrically at 330 nm.
[49]	Before sunrise, saffron was harvested from a field near Kashmar, Iran and the saffron stigmas were dried according to the method previously described by Rajabi et al. [39]. In brief, after the separation of stigmas from the flower, they were dried using a microwave oven at a power of 1000 W. Then, they were ground and passed through a sieve with a pore size of 0.421 mesh. The resulting saffron powder was poured into a dark bottle and stored inside a desiccator for further experiments. The powdered saffron and ethanol 50% (*w*/*w*) were mixed at a ratio of 1:10 and agitated using a magnetic stirrer for 2 h. Then, the extract was filtered with a filter paper (Whatman, No. 42) and concentrated using a rotary evaporator at 40 °C until 90% of the solvent was vaporized.	The content of the bioactive compounds was measured using a UV–vis spectrophotometer at 440 nm.
[50]	Powdered saffron stigmas were dissolved in boiled water and passed through a filter paper to obtain the saffron extract. The pH value was set to 7 with a phosphate buffer.	Color coordinates. In efficiency studies, absorbance was read at 240–250 nm to calculate the concentration of bioactive ingredients in nanoencapsulates.
[51]	Dried saffron stigmas (5 g) were completely ground with a porcelain mortar. The ground stigmas were extracted using methanol–water (1:1, *v*/*v*) by stirring under nitrogen at 250 rpm for 5 h. The obtained extracts were sonicated using a probe sonicator at 40 kHz and 40% of full power for 3 min, filtered, and the solvent was completely removed using rotary evaporator, transferred to Petri dishes and freeze dried for 24 h.	The analysis of the active compounds of saffron extracts was carried out using HPLC and LC-MS-MS for the identification of picrocrocin and crocin. Freeze-dried saffron extract (200 μg/mL) was redissolved in methanol–water (1:1, *v*/*v*) and filtered through a 0.2 μm (millipore) filter before analysis. Quantification of crocins was performed using calibration curves.
[52]	Saffron (*Crocus Sativa* L.) was purchased from Kashmir Kesar Pampore, Srinagar, J & K, India. Bioactives of saffron were obtained using ultrasound assisted extraction. To a powdered sample (1 g) in a flask was added 100 mL of methanol–water, 80:20 (*v*/*v*), followed by the adjustment of ultrasonicator. The temperature during extraction did not exceed 35 °C as the flask was immersed in ice. Specifically, the frequency was set at 60 Hz and the function of pulses (Pulse mode) adjusted to 5 s on, then 3 s off (Probe sonicator). The total time was 15 min for each extraction. After completing the extraction, filtration was carried out to remove solids and to obtain clear bioactives. The bioactives obtained were concentrated in a rotatory evaporator at 1/10th of the volume at 35 °C.	Total phenol content, reducing power, DPPH radical, scavenging activity. Inhibition of lipid peroxidation.
[53]	Saffron was obtained from a Persian supermarket in Montreal, Canada. For the extraction of saffron bioactive compounds, the procedure of Selim et al. [30] was followed with some modifications. Saffron powder (12 g) was mixed with 500 mL water in a dark colored bottle under continuous shaking in an incubator at 25 °C for 24 h. The extract was centrifuged at 2000× *g* for 15 min followed by filtration under vacuum. The prepared extract was freeze dried at −30 °C for 6 days and then kept in a dark colored bottle (−18 °C) until use.	The absorbance of this solution was read at 440, 330, and 257 nm using a UV-Vis spectrophotometer for crocin, safranal, and picrocrocin or particle, respectively. Absorption measurements were conducted in triplicate for each compound. The results were expressed as E^1%^_λmax_ according to [40]
[54]	as in [51]	as in [51]

Regarding saffron-sample metadata, these were either vague [40,43,50,51,53,54] or more explicit, including the name and address of the producer or supplier and often the harvest year [30,35,36,37,39,46,48,49,52].

Selim and collaborators [30] considered exhaustive extraction and used spectrophotometry to monitor crocins degradation as at that time HPLC analysis of these compounds had the drawback of a lack of standards. They also paid attention to work with saffron samples that was representative of the production of that harvest. The Cooperative of saffron in Crokos Kozanis (Greece) was the only body that had the right to collect the product from all producers and store it, after performing specific quality-control practices, at its installations as one lot.

Shakoori and Krasaekoopt [35] worked on alginate as the main supporting material and chitosan or gelatin as copolymers using an extrusion technique. The beads contained saffron extracts to be incorporated into confectionary and tea bags. They used 50% aqueous ethanol, which can support extraction of the three groups of bioactive compounds. Though no optimization study was performed for this issue, extraction was exhaustive (4 g saffron: 8 × 25 mL solvent). No yield data were reported. Saffron metadata indicated the purchase of the starting material from a well-known company but no authenticity or quality control of the starting material was mentioned. Spectrophotometric estimation of the coloring, taste and aroma strengths were carried out in the aqueous extracts of the microencapsulates and the wrong trivial name ‘crocin,’ instead of crocins, was mentioned.

The food-grade approach for the protection of crocetin through an inclusion complex with deoxycholic acid introduced by Kyriakoudi and Tsimidou [36] was based on the conformation of *trans*-crocetin, which is a linear molecule in contrast to the *cis*-isomer that tilts at C_13_ (see Table 1). A fully characterized saffron sample was used and the extraction of crocins was optimized using a response surface methodology. The researchers worked systematically to spare the precious starting material. The optimum ratio of saffron to solvent (50% aqueous methanol) was found to be (1:180 *w*/*v*). The optimization of the solvent composition for crocins and picrocrocin had been reported in a previous work [6]. The yield was satisfactory (62.7 ± 2.5 g dry extract/100 g dry stigmas). RP-HPLC-DAD was the major tool to monitor the effectiveness of the extraction process. Optical microscopy also assisted the monitoring of tissue decoloration. Charanioti and collaborators [37] worked with a mixture of saffron and beetroot extracts, which were encapsulated in maltodextrin, gum Arabic, modified starch and chitosan, with the aim of introducing natural pigments into a chewing gum. They ensured the authenticity of the saffron samples by purchasing saffron directly from the same cooperative as in [36] but they did not check its initial content in bioactive compounds. Moreover, no extraction yield is reported, whereas results for ‘crocin’ degradation during storage (dark, 40 °C, 10 weeks) were expressed semiquantitatively using the ISO3632-2 spectrophotometric approach [2]. Rahabi and collaborators [39] worked with fresh saffron stigmas picked from the field and processed further in the laboratory at different conditions, and observed higher values of all three groups of the bioactive compounds when microwave heating at 1000 W was applied. Saffron extracts were prepared using 50% aqueous ethanol, which is expected to extract a higher amount of hydrophobic compounds such as safranal. Spectrophotometric assessment at 257, 330 and 440 nm was according to a past publication [58], in which factors influencing absorbance values and means for improvement of the relevant ISO 3632-2 approach [2] were discussed. Four years later, the same group [49] used another system for saffron bioactive compounds following a similar approach regarding the starting material and extraction method, whereas they used only absorption data at 440 nm to monitor efficiency.

Esfanjani and collaborators used the same plant material obtained from Torbat Heydariyeh farms (Khorasan-e-Razavi, Iran) in their two publications [40,43]. This was evidenced by the fact that the percent content values for crocins, picrocrocin and safranal were the same in the analyzed aqueous extracts. They followed the same extraction procedure in their two publications, but cited different sources for it. The full description of the extraction protocol is given in Table 2. The extracts were then examined using the ISO 3632 spectrophotometric method for a tentative estimation of the coloring strength, flavor and aroma of saffron [2]. This analytical method—as mentioned before—is used to give the respective E^1%^ _λmax_ values, which are useful for the commercial categorization of saffron. However, it is not a precise method for the determination of the actual concentrations of the bioactive compounds. It should also be pointed out that safranal, which is an in-vivo antioxidant, is not expected to be transferred quantitatively in aqueous extracts [8]. Consequently, this analytical process can be applied tentatively only for the crocins and picrocrocin. Moreover, it is impossible to find two batches of saffron that have, simultaneously, the same coloring strength, bitterness, and aroma E^1%^ _λmax_ values. Thus, the encapsulation process in multiple emulsions based on the pectin–whey protein complex proposed by [40] and [43] is difficult to duplicate regarding this factor.

Kyriakoudi and Tsimidou [46] worked in a more systematic way using saffron samples checked for authenticity both analytically and administratively, as the material provided by the Greek Saffron Producers Cooperative (Kozani, Greece) was certified by an externally accredited laboratory. Extraction of crocetin esters and picrocrocin was optimized using the response surface methodology to reduce the significant cost of the process and avoid a high-cost starting material. Ultrasound-assisted extraction was employed to accelerate the process [6,36]. This technology is currently an established extraction technique of phytochemicals at industrial scale [60]. The percent extraction yield at the optimum conditions for the recovery of total crocetin (methanol–water, 1:1, *v*/*v*; saffron–solvent ratio = 1:182, *w*/*v*; sonication duration = 30 min, duty cycles of sonication (active interval) (s) (0.2 s on/0.8 s off)) was 62.7 ± 2.5 (*n* = 3). No further extraction circles were found to be necessary on the basis of spectrophotometric data and optical microscopy observation of the tissues. Picrocrocin levels under the optimum conditions for crocins extraction were 11 ± 2 instead of 12 ± 1 mg kg−1 dry stigmas for its own extraction optimum (0.44% methanol, 30 min, 0.6 s). Consequently, the former conditions were adopted as optimum for both categories of apocarotenoids. The authors pointed out that by using raw materials sparingly, energy cost due to the implementation of ultrasounds was compensated to a certain extent. Then, the researchers [6] followed a precise RP-HPLC- diode array procedure using laboratory-prepared standard materials of a known composition to fully characterize the content of crocins and picrocrocin in both the aqueous saffron extracts and the obtained nanoencapsulates in maltodextrin using the Büchi B-90 Nano Spray Dryer. The authors used two external calibration curves, one with *trans*-crocetin di *(β*-D-gentiobiosyl) ester (y = 28,296.06x – 109,972.40, R^2^ = 1.00 in the range 9–455 ng/10 μL injected volume, *n* = 7) and another for picrocrocin (y = 27,653.09x – 170,026.79, R^2^ = 1.00 in the range 10–295 ng/10 μL injected volume, *n* = 5). The standard materials were laboratory-isolated. *Trans*-crocetin di *(β*-D-gentiobiosyl) ester was isolated by semi-preparative RP-HPLC and its purity (97%) was checked (a) chromatographically by RP-HPLC-DAD in the range of 200–550 nm and calculated as the percentage of the total peak area at 440 nm and (b) by proton nuclear magnetic resonance spectroscopy (1H NMR) at 300 MHz. Picrocrocin was isolated according to Sánchez et al. [59]. Purity of isolated picrocrocin (91%) was checked by RP-HPLC at 250 nm and by 1H NMR (300 MHz, CD_3_OD), as reported by Kyriakoudi and collaborators [6]. These analytical protocols are very useful to check the purity of either laboratory-prepared or commercial standards. The nanoencapsulation procedure described by Kyriakoudi and Tsimidou [46] is a repeatable process regarding the characteristics of the starting material, as the ratio of core–wall material was calculated on a weight basis. In the stability studies of the nanoencapsulates, the same authors monitored the losses of the bioactive compounds under study by spectrophotometry in the region 200–600 nm and quantified them as total crocins and picrocrocin using the corresponding calibration curves (y = 0.52x − 0.031; R^2^ = 0.99; 1–50 mg/L, *n* = 7 and y = 0.459x − 0.0201; R^2^ = 1; 1–50 mg/L, *n* = 6). In this view, absolute values of the encapsulated material were determined through stability studies in the dark (60 °C) and under gastrointestinal conditions.

Dehcheshmeh and Fathi [48] published work on the production of core-shell nanofibers from zein and tragacanth for the encapsulation of saffron extract and although they work with aqueous saffron extracts, they chose to monitor the release of safranal and not of crocins or picrocrocin for saliva, water, gastric and intestinal media using different mathematical models. However, the authors do not justify their preference for using safranal, which is poorly transferred in the aqueous extracts and its maximum wavelength is at 308 nm and not at 330 nm. Building models upon E ^1%^ values probably leads to questionable results, even if figures obtained seem to be satisfactory.

Hadavi and collaborators’ [50] approach includes basic weaknesses and inaccuracies as their starting material was purchased from a well-known supplier (Novin Saffron Co., Mashhad, Iran) but was not further checked for its quality characteristics. Moreover, the method for the preparation of the saffron extract is not clearly described and raises many questions as to how and why they chose to dissolve powdered saffron stigmas in boiled water and pass it through a filter paper (Whatman, No. 42) before further use. It is not also clear if they dried this extract, or not, before mixing with the encapsulation nanoliposomes ingredients, though they report w/w amounts of saffron in the mixture for encapsulation. Spectrophotometric evaluation for the content of bioactive ingredients is vague (which ones were the target?) and seems inaccurate as the range 240–250 nm is not appropriate for any of the saffron bioactive groups of compounds. Optimization studies using the response surface methodology seem pointless because the outcomes cannot be further used as no objective determination of saffron bioactive compounds was carried out.

In two publications, Najafi and collaborators [51,54] worked with saffron material purchased from Jamshidi Marandi (Khorasan-e-Razavi, Iran, harvest 2020) without further contact information for the producer. They prepared saffron extracts in methanol–water (1:1, *v*/*v*) but no reference is given for their choice. The complete extraction process is shown in Table 2. The obtained extraction yield (g extract/100 g of stigmas), 55 ± 3%, was lower than that reported in [36]. Nahafi and collaborators followed a similar approach to that reported by Kyriakoudi and Tsimidou [46] for identifying and quantifying crocins in the saffron extracts and in the other encapsulation studies. No due credit to relevant publications was found in their reference list. ‘Crocin-4′ with purity of % 98 (Biopurify Phytochemicals Ltd., Sichuan, China) was used as an external standard. The equation used for quantification of ‘crocin-4 ‘(i.e., *trans*-crocetin di (β-D-gentiobiosyl) ester) and other crocins was y = 156331X, R^2^ = 0.999. Identification of picrocrocin was performed using LC-MS, whereas its quantification was carried out using a regression equation from the literature (y = 1,952,830x − 3808.1) for Italian saffron [61]. The latter cannot be considered as an appropriate analytical methodology and jeopardizes the input of the chosen sophisticated analytical technique. Nevertheless, their effort to incorporate nanoencapsulated saffron extracts into edible films incorporated the concept into work using accurate identification and quantification protocols.

Gani and collaborators [52] encapsulated saffron and sea-buckthorn bioactives to utilize them for the development of low-glycemic baked products for the world’s increasing diabetic population. They mention where they bought saffron as a starting material but no further examination of its content or its actual bioactive components is performed throughout the study. Total phenol content and antioxidant activity tests were used instead, which suit more the examination of buckthorn bioactive compounds, as saffron aqueous extracts are not rich in radical scavengers such as phenolic antioxidants and tocopherols [62].

### 3.2. Commercial ‘Crocin’ Products or Other Saffron Bioactive Compounds as Starting Material

Table 4 presents the published work on nanoencapsulation efforts for saffron bioactive compounds with, as starting material, commercial or laboratory-prepared products or isolated compounds.

**Table 4:** En(nano)capsulation approaches for saffron bioactive antioxidants with focus on the characteristics of commercial or laboratory prepared products as the starting material, extraction and analytical methods for their isolation and determination.

Reference	Sample Preparation	Analytical Methods
crocins and crocetin		
[33]	Crocetin reference material (purity up to 96%) was laboratory prepared.	Spectrophotometrically at 421 nm (methanol as solvent) using the extinction value 252.0 for crocetin and appropriate dilution factor for quantification.
[38]	‘Crocin’ was purchased from Sigma-Aldrich Chemical Co. (St. Louis, MO, USA). Crocin (0.06% (*w*/*v*)) was placed into alginate solution.	The ‘Crocin’ content was analyzed using a UV–Vis spectrophotometer at 440 nm.
[41]	‘Crocin’ (MW: 976.96 g/mole, Purity ≥ 95%) was purchased from Sigma–Aldrich Co. (St. Louis, MO), ‘Crocin’ (10 wt.% solution in water).	Tristimulus values (L*, a*, b*) of color coordinates.
[42]	‘Crocin’ was purchased from Sigma-Aldrich Co. (St. Louis, MO) 0.2% ‘Crocin’ aqueous solution.	Released ‘Crocin’ was analyzed using a UV–Vis spectrophotometer at 440 nm.
[44]	as in [38].	as in [38].
[45]	Crocetin was extracted from plant *Crocus sativus* L. based on the method represented in Iran patent no. 84459.	The concentration of crocetin was determined using a UV spectrophotometer at 430 nm and a crocetin standard curve.
[47]	‘Crocin (0.1 g) was solved in 5 mL of water heated to 50 °C before further use. Crocetin (purity ≥ 90%) was obtained using ‘crocin’ hydrolysis according to a patented method [28].	The amount of crocin and crocetin released was determined as reported elsewhere [44]. Calibration curves for crocetin and ‘crocin’ were performed in the concentration range of 10–100 mg/mL (*n* = 6).
[55]	1 mL of a 1050 µg/mL ‘crocin’ (Bu Ali Research Institute, Mashhad, Iran) solution was mixed with alginate solution using a magnetic stirrer at 500 rpm for 20 min before further use.	A UV spectrophotometric method at 440 nm was used. Calibration curves were used.
safranal		
[34]	Safranal (Fluka, Spain).	Quantitative determination was carried out by RP-HPLC at 308 nm. Safranal and liposomal safranal was dissolved in methanol. External calibration curve was used.

Zhou and collaborators [33] tested the performance of three wall materials (beta-cyclodextrin, gum Arabic and maltodextrin) for the oxidative protection of crocetin by spray drying the first time with three wall materials (beta-cyclodextrin, gum arabic and maltodextrin). No method for the isolation of crocetin in the laboratory or purity test results was presented. Deterioration kinetics of laboratory-prepared crocetin was monitored spectrophotometrically. An effort for the semiquantitative estimation of the crocetin content in the encapsulates was based on the use of an extinction value according to an approach used in the past for β-carotene.

Malaekeh-Nikouei and collaborators [34] used liposomes as a carrier of commercially available safranal to improve its potential anti-tumor effect using different cell lines. Analysis of safranal was accomplished using a quantitative RP-HPLC at 308 nm and using an external calibration curve.

Rahaiee and collaborators [38,44], as well as Mehrnia and collaborators [41,42], purchased ‘crocin’ (MW: 976.96 g/mole, purity ≥ 95%) from Sigma–Aldrich Co. (St. Louis, MO, USA) without further checking of the composition of the commercial standard. As can be deduced from the introduction of the publications mentioned above, they consider that the commercial ‘crocin’ product is the *trans*--crocetin di *(β*-D-gentiobiosyl) ester, which they name ‘crocin’. This term is not precise and can cause further confusion if the researchers do not use official nomenclature for the chemical compounds and do not check the exact concentration of the commercial products they employ. Therefore, regarding publication [41], the reported optimum concentration level of ‘crocin’ (0.1%), stirring speed, 700 rpm, and ambient temperature for the spontaneous encapsulation has value only as a methodological proposal to produce nanoscale emulsions at low energy cost and with the minimum use of surfactants. The measurement of color coordinates (L*, a*, b*) was the sole method related to the presence of apocarotenoids in the study. In the second publication [42], the purity of the commercial product is not mentioned; the researchers used 0.2% ‘crocin’ for encapsulation whereas released crocin was determined spectrophotometrically at 440 nm; and its content possibly expressed as E^1%^ _λmax_, as no calibration curve is mentioned for quantification. The two works have drawbacks regarding the control of the starting material and the accurate determination of the amounts of encapsulated bioactive compounds, irrespective of the encapsulation approaches used. Obviously, the researchers were focused only on the crocins and not on picrocrocin. Rahaiee et al. [38,44] did not mention the purity of the commercial product, although this was purchased from Sigma–Aldrich Co. ‘Crocin’ was monitored spectrophotometrically at 440 nm, without further explanation of any type of quantification of the biodegradable nanoparticles of chitosan-alginate in release studies. The reference they give is misleading as far as it concerns the spectrophotometric estimation of crocins content [63]. Moreover, it is not justified why, in the introductions of the two publications, they refer extensively to all saffron bioactive compounds when their experimental work had to do only with crocins.

Puglia and collaborators [47] also used ‘crocin’ (batch number: BCBT4979) from the same company, which then also served as a starting material for the preparation of crocetin (purity > 90%) according to a hydrolysis and purification process reported in an international patent [64]. Nevertheless, the process is not detailed concerning verifying whether and how purity control was performed and the narrative of the patent is not informative enough. Similar observations apply for another publication [45], which refers to the isolation of crocetin using an Iranian patent that is not accessible and lacks further details for the isolation of the crocetin or its purity control. It is somewhat unusual that the authors preferred a patented unclear description and did not follow the crocetin precipitation procedure described clearly and more recently by Kyriakoudi and Tsimidou [36], who performed purity control using UV-Vis spectroscopy, RP-HPLC-DAD, FT-IR and NMR spectroscopy. According to Kyriakoudi and Tsimidou [36], RP-HPLC data showed that the apocarotenoid mixture was ~98% pure and consisted of *trans-* and *cis-*isomers, with the former being ~89%. Moreover, even if a calibration curve is reported by [45], they do not give any equation or range of the standard solutions used. Puglia and collaborators [47] employed only spectrophotometry at 440 nm to monitor the presence of the two apocarotenoids according to [44]. No quantification means were detailed, meaning that their procedure includes serious weaknesses regarding this issue.

Nasrpour and collaborators [55] purchased ‘crocin’ from the Bu Ali Research Institute (Mashhad, Iran). In their publication, the authors use the name ‘crocin’ without any mention of the chemical structure of the corresponding compound. No data for the purity of the starting material or purity control is reported. For quantification of crocin in the supernatant after release from the nanoencapsulates, a UV spectrophotometric method at 440 nm was used. The method was linear in the range of 0.125–12.5 µg/mL (R^2^ = 0.999; y = 0.078x + 0.0051). The limit of quantitation (LOQ) was 0.125 µg/mL (cv < 5%).

## 4. Gaps and Proposals Regarding the Starting-Material Choice and Analysis

Saffron is a complex natural matrix. A deeper understanding of this matrix as a commodity and also as a source of bioactive antioxidants is necessary when selecting a saffron sample as a starting material in encapsulation studies. The literature on encapsulation is accumulated but all researchers and—possibly some reviewers—do not seem to be aware about the importance of providing metadata for the provenance of the material, harvest date, authenticity and quality control of the saffron sample, or details on the purity of commercial samples. The term ‘crocin’ is misleading, not chemically correct and authors should be precise in descriptions because, in fact, they use mixtures of crocins even though one of them, the *trans-* crocetin di *(β*-D-gentiobiosyl) ester, prevails in concentration. Not to forget that saffron samples and commercial standards deteriorate and are prone to oxidation and isomerization. Application of nanoencapsulation technologies further affect the stability of sensitive compounds [65,66]. Storage conditions and gastrointestinal environment have a negative effect on the stability and bioavailability of all saffron bioactives, respectively [46,55]. Bioactivity is related to specific compounds, meaning that the introduction of chromatographic procedures for the separation, identification and quantification of the relevant ones is necessary. UV-Vis spectrophotometry without the use of calibration curves for each bioactive compound should be avoided as an analytical tool in these studies. Arbitrary methods of assessment of coloring strength, bitterness and aroma [2] are not appropriate in nanoencapsulation studies. Even if a standard is used, the information obtained concerns the total crocins concentration and the results should be expressed accordingly. Aqueous extracts of saffron can be used as sources for crocins and picrocrocin as core compounds. Crocetin derived from the hydrolysis of crocins needs extensive characterization before encapsulation. Once again, spectrophotometry cannot distinguish between *trans-* and *cis-*isomers. No work was found for picrocrocin nanoencapsulation, whereas only one was dedicated to safranal. Introduction of statistical tools in experimental designs cannot compensate for weaknesses such as the abovementioned. Nanoencapsulation is an emerging and attractive field for a wide array of sensitive compounds and enzymes [67]. The literature is accumulated quickly and new technologies and wall materials are tested continuously, so it is important to avoid certain loopholes such as those discussed in the present review.

## Figures and Tables

**Table 1:** Major bioactive apocarotenoids of saffron.

Crocins (*trans-* and *cis-*forms, left and right side, respectively)	
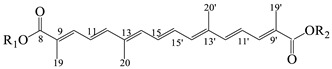	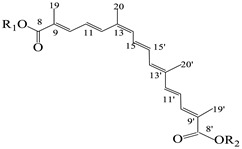
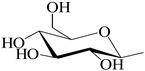 A	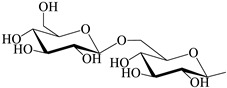 B
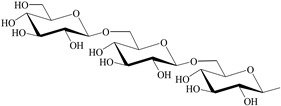 C	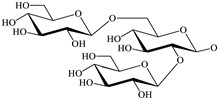 D
Group	Chemical compound
R_1_ = R_2_ = B	*trans*-crocetin di(*β*-D-gentiobiosyl) ester
R_1_ = C (or D), R_2_ = B	*trans*-crocetin tri(*β*-D-glucosyl)-(*β*-D- gentiobiosyl) ester (or *trans-*crocetin *(β*-D- gentiobiosyl)-(*β*-D-neopolitanosyl) ester)
R_1_ = B, R_2_ = A	*trans-*crocetin (*β*-D-gentiobiosyl)-(*β*-D-glucosyl) ester
R_1_ = R_2_ = A	*trans*-crocetin di (*β*-D-glucosyl) ester,
R_1_ = B, R_2_ = H	*trans*-crocetin (*β*-D- gentiobiosyl) ester,
R_1_ = A, R_2_ = H	*trans*-crocetin (*β*-D-glucosyl) ester,
R_1_ = R_2_ = H	crocetin
R_1_ = R_2_ = CH_3_-	dimethyl ester of crocetin
Other major bioactive compounds	
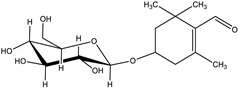 4-(*β*-D-glucopyranosyloxy)-2,6,6-trimethyl-1-cyclohexene-carboxaldehyde) (picrocrocin)	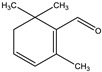 trimethyl-1,3-cyclohexadiene-1-carboxaldehyde) (safranal)

**Table 2:** Annual number of publications since 2000 using as keywords saffron and authenticity and saffron and health in the title, abstract, or keyword fields (Scopus search carried out in January 2023).

Year	Publications No			Publications No	
	A *	B **	Year	A *	B **
2022	8	62	2010	0	5
2021	3	49	2009	0	4
2020	6	31	2008	0	3
2019	4	26	2007	0	2
2018	4	29	2006	0	0
2017	8	19	2005	0	0
2016	4	20	2004	1	3
2015	3	13	2003	0	0
2014	0	11	2002	0	1
2013	1	10	2001	0	1
2012	0	7	2000	1	1
2011	2	10			

* A: saffron and authenticity; ** B: saffron and health.

## Data Availability

The data is contained within the article.
